# Significant Adhesion Reduction with 4DryField PH after Release of Adhesive Small Bowel Obstruction

**DOI:** 10.1055/s-0039-1687857

**Published:** 2019-05-10

**Authors:** Mouhannad Ahmad, Fabio Crescenti

**Affiliations:** 1 Aller-Weser-Klinik, Verden (Aller), Germany

**Keywords:** ileus, intestinal obstruction, modified polysaccharide, barrier gel, adhesion prevention

## Abstract

**Background** Peritoneal adhesions reoccur in up to 100% of cases, possibly causing complications like pain, secondary female infertility, and small bowel obstruction. The latter has a mortality rate of up to 15% during hospitalization. This study investigates if recurrence of peritoneal adhesions can be prevented by prophylactic use of the starch-based medical device 4DryField.

**Methods** The course of 40 patients with surgery for intestinal obstruction and, partially, second intervention was analyzed. In both operations, adhesion severity and extent were scored 0 (no adhesions) to III (massive/dense and vascular adhesions) and 0 (no adhesions) to III (extensive, covering more than approximately 25 × 25 cm), respectively. To prevent recurrence of adhesions all patients were treated with 4DryField gel (60 mL saline solution per 5 g powder), evenly distributed on the whole impaired intestine (including anastomoses) before abdominal closure. Follow-up was up to 1.5 years in a 3 to 6 months' interval.

**Results** Eight patients had relaparotomies on postoperative days 1 to 155. In the first operation, median adhesion severity score was III, median adhesion extent II. In redo-surgeries, significantly lower scores were detected (median adhesion severity: 0, *p* = 0.0003; median adhesion extent: 0, *p* = 0.0009). No adverse events related to the product were observed. One patient had later redo-surgery in another hospital due to recurrence of adhesions, one patient suffered from flatulence. All other patients were free of adhesion-related symptoms during follow-up.

**Conclusion** Based on the high severity of diseases and the significant reduction of adhesion severity and extent in redo-surgeries, 4DryField gel is a promising adjunct for adhesion prevention in bowel surgery. The favorable results should be confirmed in prospective randomized trials.

Adhesions are abnormal fibrous tissue bands that develop between damaged neighboring mesothelial surfaces normally not attached to each other via a fibrin matrix which is gradually filled with different cell types. 1 In other words, adhesions are “scars” of the peritoneum. They result from the biochemical and cellular response occurring in an attempt to repair the peritoneum. 2 The formation of adhesions at the molecular level involves a complex interaction of cell adhesion molecules, cytokines, growth factors, neuropeptides, and several other factors secreted by cells near the traumatized area. 3 In this process, the early balance between fibrin deposition and degradation seems to be the critical factor in adhesion formation. 2 In general, peritoneal adhesions develop after more than 90% of operations in the abdominal cavity. 4 5 6 7 8 Postoperative adhesions are the most frequent cause for intestinal obstruction, a severe complication often necessitating surgical treatment. 9 Obstruction can cause bowel necrosis and is reported to have a mortality rate of up to 15% during hospitalization. 10 Vrijland et al 11 reported that following surgical adhesiolysis adhesions reoccur in up to 100% of cases confirmed with second-look laparoscopy and they reoccur at the sites of former adhesiolysis. 12 This might explain the high risk of reoccurrence of small bowel obstruction (SBO) as reported by Suter et al 9 and Barkan et al. 13 In addition, with every episode relapse becomes more frequent and the intervals between obstructions become shorter. 13 Moreover, adhesiolysis results in longer operation times with inadvertent enterotomies in 6 to 19% of cases and the course of recovery is prolonged and more complicated. 14 15 16 Thus, adhesion prevention is a major concern after surgical adhesiolysis and release of adhesive bowel obstruction.

The treatment of adhesion-related complications contributes substantially to health care costs. In the U.S. adhesiolysis-related costs were estimated to be as much as U.S. $2.3 billion in 2005, 17 corresponding to approximately U.S. $600 million translated to Germany. In a smaller Swedish study comprising 102 patients operated for SBO, there were 273 episodes of SBO with 237 readmissions and 47% of episodes resulting in further surgery within a mean follow-up of 14 years. Extrapolated to the whole of Sweden, the annual cost of adhesion-related complications was estimated as €40 to 60 million, 18 corresponding to approximately €330 to 500 million translated to Germany. Thus, effective adhesion prevention can substantially contribute to avoid secondary health care costs.

In reviews by Korell 19 and Crowe and Trew, 20 advantages and disadvantages of several adhesion prevention devices were evaluated. Those and other authors concluded that the suitability of devices was limited for the following reasons: drains cannot be inserted, laparoscopic application is difficult, presence of blood is rendering them inefficient, and their use in bowel surgery is not possible. 19 20 21 22

In this study, the starch-based medical device 4DryField PH (4DF; PlantTec Medical GmbH, Lüneburg), certified for hemostasis and adhesion prevention, which can easily be applied laparoscopically, was used in a prophylactic manner in patients undergoing surgery for adhesive SBO. The 4DF is applicable for bowel surgery and allows insertion of drains. In addition, no adverse events have been reported. As powder, 4DF effectively provides hemostasis, particularly in diffuse bleeding. 23 24 25 When dripped or mixed with saline solution, the powder particles form a barrier gel, shown to be highly effective in preventing adhesions in animal experiments, as well as gynecological and visceral surgery. 23 24 26 27 28 29 30 31

## Materials and Methods

This retrospective study was approved by the Ethics Committee of the Hannover Medical School (no. 2773–2015) and has therefore been performed in accordance with the ethical standards laid down in the 1964 Declaration of Helsinki and its later amendments. All procedures followed were in accordance with the ethical standards of the responsible committee on human experimentation (institutional and national) and with the Helsinki Declaration. The observational trial comprises 40 consecutive patients (28 females, 12 males) with surgery for bowel obstruction due to adhesions operated between 2014 and 2016. Operation reports, final reports of hospitalization, follow-up reports, routine laboratory tests, and reports of subsequent hospitalizations and treatments in other institutions were evaluated. Patients' data like age, sex, height, weight, body mass index (BMI), previous surgeries, American Society of Anesthesiologists (ASA) score, diagnosis, operation time, type of operation, and duration of hospitalization were recorded. In addition, routine laboratory parameters, including hemoglobin, hematocrit, leucocytes, thrombocytes, C-reactive protein (CRP), and blood glucose values were evaluated and statistically compared with those of a cohort of 10 patients with the same type of operation but no application of 4DF.

The severity of adhesions was graded 0 = no adhesions; I = filmy adhesions, blunt dissection; II = strong adhesions, sharp dissection; or III = very strong vascularized adhesions, sharp dissection, damage hardly preventable following Coccolini et al. 32 The extent of adhesions was scored 0 = no adhesions; I = localized (covering an area smaller than approximately 15 × 15 cm); II = moderate (covering between approximately 15 × 15 cm and 25 × 25 cm); or III = extensive (covering more than approximately 25 × 25 cm) modified from The American Fertility Society, 33 Brown et al, 34 and Trew et al. 35 Adhesion scores were statistically compared between first and redo-surgeries.

To prevent recurrence of peritoneal adhesions, all patients were treated with 4DF gel. A gel made of either 5 g 4DF powder and approximately 60 mL saline solution, 10 g 4DF powder, and approximately 120 mL saline solution or 15 g 4DF powder, and approximately 180 mL saline solution was used, depending on the extent of the wound areas. The gel was evenly distributed on all affected surfaces of the intestine and in the peritoneal cavity. Patients were followed up for up to 1.5 years, including outpatient or in house visits within the first 3 months after discharge and via telephone interviews in 3 to 6 months' intervals in the later course. *p* -Values were determined using an unpaired two-tailed *t* -test for normally distributed data and a two-tailed Mann–Whitney test for not normally distributed data. In addition, a log-rank test (Mantel–Cox test), typically used when the measurement is the time to an event, was executed to compare the operation times. A *p* -value below 0.05 was considered statistically significant.

## Results

The patients were between 21 and 92 years old (mean = 63.5 years), the BMI was between 17.8 and 37.6 kg/m ^2^ (mean = 26.1 kg/m ^2^ ), the average ASA score was 2.5. Overall, 20 patients (50%) could be treated solely laparoscopically in the first operation of the present study, while 10 patients (25%) necessitated conversion to open surgery. Reasons for conversion to laparotomy were accompanying diseases, particularly dense adhesions, or failure to identify the obstructing adhesion. Ten patients (25%) had open surgery from the beginning due to the severity or acuteness of the disease. In total, 90% of the patients had had previous surgery before the first intervention of the present study ( Table 1 ). Of the 20 patients with laparoscopic first surgery, two had had no previous intervention but at the time of the first surgery of the present study one had an acute cholecystitis and one had an SBO. Of the 10 patients with conversion, all had had previous interventions, whereas two of the 10 patients with open surgery from the beginning had had no previous intervention. Both these patients had an intestinal obstruction due to volvulus at the time of the first surgery of the present intervention. The overall mean operation time was 155.3 minutes (range, 46–355 minutes). There was no major intraoperative blood loss as administration of blood or blood products did not become necessary. In 11 patients with open intervention ( *n* = 8) or conversion from laparoscopic to open surgery ( *n* = 3), hemoglobin concentrations fell below 10 g/dL postoperatively with minimum values of 6.3 to 9.9 g/dL. The mean postoperative laboratory parameters of the whole cohort of 40 patients treated with 4DF were statistically compared with those of a cohort of 10 patients with the same type of operation but no application of 4DF. The comparability of both patient groups was ensured by statistical comparison of age, BMI, and ASA score. Mean age was 63.5 years in the 4DF versus 55.5 years in the control cohort ( *p* = 0.2769), mean BMI was 26.1 versus 26.0 kg/m ^2^ ( *p* = 0.9462), mean ASA score was 2.5 versus 2.0 ( *p* = 0.0528). Since there were no significant differences, the comparability of the patient groups was given. The mean postoperative hemoglobin level (11.5 vs. 13.1 g/dL; *p* = 0.0031) and the mean postoperative hematocrit level (34.0 vs. 37.7%; *p* = 0.0128) were significantly lower in the 4DF group as compared with the untreated patients. The mean postoperative thrombocyte level (268.4 vs. 245.4; *p* = 0.5446), the mean postoperative leucocyte level (9.8 vs. 8.0/nL; *p* = 0.2355), the mean postoperative blood glucose level (127 vs. 153 mg/dL; *p* = 0.6176), and the mean postoperative body temperature (37.2 vs. 37.0°C; *p* = 0.1221) did not differ significantly between both groups. The mean postoperative CRP level was significantly higher in the 4DF group as compared with the untreated patients (11.5 vs. 3.3 mg/dL; *p* < 0.0001). In addition, the operation time was significantly longer in the 4DF group as compared with the untreated patients (mean = 155.3 vs. 70.1 minutes; *p* = 0.0001). All increases and decreases were temporary. The temporary rise of the CRP levels in patients with 4DF application usually peaked on postoperative days 2 or 3 without accompanying leukocytosis or rise of body temperature. An improper postoperative rise in blood glucose levels was not observed, not even in two patients with known diabetes.

**Table 1:** Data and clinical outcome of all 40 patients, grouped by type of 1st operation

**Laparoscopic adhesiolysis (** ***n*** ** = 20)**																			
**Mean age, y (range)**	**Previous surgeries** (%)			**Adhesion severity at 1st OP** (%)				**Adhesion extent at 1st OP** (%)				**Adhesion severity at 2nd OP** (%)				**Adhesion extent at 2nd OP** (%)			
65.7 (33–92)	None	Lap.	Open	0	I	II	III	0	I	II	III	0	I	II	III	0	I	II	III
	10	25	65	0	10	30	60	0	40	25	35	80	0	20	0	80	0	20	0
**Conversion (** ***n*** ** = 10)**																			
**Mean age, y (range)**	**Previous surgeries** (%)			**Adhesion severity at 1st OP** (%)				**Adhesion extent at 1st OP** (%)				**Adhesion severity at 2nd OP** (%)				**Adhesion extent at 2nd OP** (%)			
62.9 (31–86)	None	Lap.	Open	0	I	II	III	0	I	II	III	0	I	II	III	0	I	II	III
	0	20	80	0	0	0	100	0	0	50	50	100	0	0	0	100	0	0	0
**Open adhesiolysis (** ***n*** ** = 10)**																			
**Mean age, y (range)**	**Previous surgeries** (%)			**Adhesion severity at 1st OP** (%)				**Adhesion extent at 1st OP** (%)				**Adhesion severity at 2nd OP (%)**				**Adhesion extent at 2nd OP (%)**			
59.7 (21–81)	None	Lap.	Open	0	I	II	III	0	I	II	III	0	I	II	III	0	I	II	III
	20	10	70	0	10	20	70	0	10	40	50	100	0	0	0	100	0	0	0
**Total (** ***n*** ** = 40)**																			
**Mean age, y (range)**	**Previous surgeries (%)**			**Adhesion severity at 1st OP (%)**				**Adhesion extent at 1st OP (%)**				**Adhesion severity at 2nd OP (%)**				**Adhesion extent at 2nd OP (%)**			
63.5 (21–92)	None	Lap.	Open	0	I	II	III	0	I	II	III	0	I	II	III	0	I	II	III
	10	20	70	0	7.5	20	72.5	0	22.5	35	42.5	87.5	0	12.5	0	87.5	0	12.5	0

Abbreviations: lap., laparoscopic; OP, operation; y, years.

*Note* : Adhesion scores at first and second operation were modified from Coccolini et al, 32 as well as The American Fertility Society, 33 Brown et al, 34 and Trew et al. 35

*Note* : Adhesion severity was classified 0 = no adhesions; I = filmy adhesions, blunt dissection; II = strong adhesions, sharp dissection; III = very strong vascularized adhesions, sharp dissection, damage hardly preventable.

*Note* : Adhesion extent was classified 0 = no adhesions, I = localized (covering an area less than approximately 15 × 15 cm), II = moderate (covering between approximately 15 × 15 cm and 25 × 25 cm), III = extensive (covering more than approximately 25 × 25 cm).

In the first operation of the present study, the median adhesion severity score of all 40 patients treated with 4DF was III and the median adhesion extent was II. In 29 patients (73%), peritoneal adhesions were massive with a severity score of III. All these patients had extensive adhesions mostly between the small bowel loops and the abdominal wall in the lower abdomen. In four patients, the lesser pelvis was affected as well, in one also the colon. In nine patients, adhesiolysis was accompanied by appendectomy ( *n* = 2), cholecystectomy ( *n* = 2), fistulectomy ( *n* = 1), hemicolectomy ( *n* = 2), partial small bowel resection with anastomosis ( *n* = 2), or rectum extirpation ( *n* = 1).

Postoperatively, 18 patients experienced temporary pain in the first 3 days after surgery. None of the patients with an operation time of ≤ 60 minutes had any postoperative complications. Five patients had a wound dehiscence (one after laparoscopic intervention, three following conversion from laparoscopic to open surgery, and one subsequent to open surgery). The latter patient, as well as one of the patients with conversion developed an accompanying infection caused by the skin bacteria *Staphylococcus aureus* . One patient with conversion from laparoscopic to open surgery had a paralytic ileus which was treated conservatively. One patient with open surgery, exorbitant adhesions, and partial small intestine resection developed a subphrenic abscess. The abscess was treated with pigtail drainage and antibiotics.

**Fig. 1:**
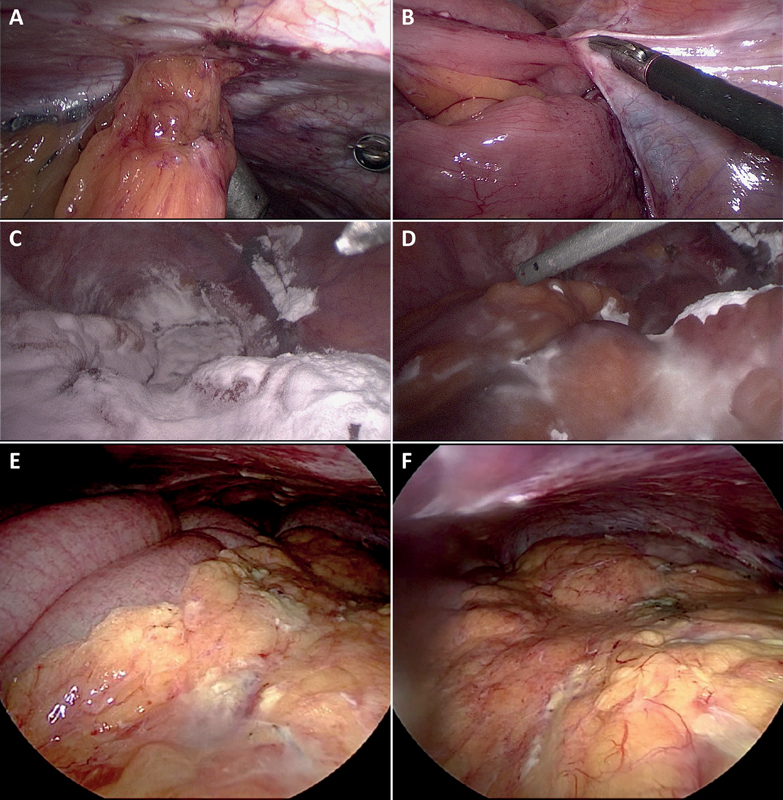
Representative images of the first ( **A** – **D** ) and second ( **E, F** ) operation of the same female patient with laparoscopic adhesiolysis. ( **A, B** ) aspects of intestinal adhesions being lysed during the intervention, ( **C** ) application of 4DryField PH after extensive adhesiolysis, ( **D** ) transformation of the powder into barrier gel by dripping with saline solution, ( **E, F** ) aspects of the operative site during the second operation revealing no adhesion formation.

In the postoperative course, eight patients required reinterventions between postoperative days 1 and 155 (mean = 28 days). In the reoperations no remnants of 4DF were detected. All eight patients had had previous surgeries before the present study (seven patients had had open surgeries, one laparoscopic surgery). The first operation of these patients in the present study was laparoscopy in five patients, conversion in two, and open surgery in one. The five patients with laparoscopic first operation were reoperated due to acute abdomen (distended small bowel and possible perforation detected, sigma diverticula sewn over during relaparotomy), abdominal pain, appendicitis, peritonitis caused by small bowel perforation, and suspected mechanical obstruction (which was not confirmed during relaparotomy), respectively. The two patients with conversion in the first operation were reoperated due to wound dehiscence (epifascial vacuum seal applied) and abdominal pain (partial resection of the ileum and vacuum seal performed), respectively. The patient with open first operation was reoperated due to abdominal compartment syndrome (this patient died after the reintervention). Only one of the patients revealed recurrence of adhesions in the reintervention after 4DF application when being reoperated for abdominal pain in another hospital. All other patients were free of adhesions in the reinterventions after 4DF application ( Fig. 1 ). One of the reoperated patients did not show adhesions in redo-surgeries on postoperative days 13 (for treatment of a wound dehiscence with epifascial vacuum seal) and 21 (for closure of the abdominal wall). However, this patient revealed extensive and severe adhesion formation in a third reintervention on postoperative day 25 (due to wound dehiscence) after no 4DF had been applied in the second reintervention. The reasons for the strong adhesion formation found in the third reintervention on postoperative day 25 likely were fasciitis and the lack of a 4DF application in the previous reintervention on postoperative day 21. In general no adverse events related to 4DF were observed.

Overall, in the first operation the median adhesion severity score was III, the median adhesion extent II. In the redo-surgeries, significantly lower scores were detected (median adhesion severity: 0, *p* = 0.0003; median adhesion extent: 0, *p* = 0.0009). Data and clinical outcome of all 40 patients, grouped by type of first operation, can be found in Table 1 .

During follow-up after hospitalization, none of the 40 patients had recurrent obstruction and no adverse events related to 4DF were observed. One patient had recurrent abdominal pain and changed to another hospital where she had redo-surgery with adhesiolysis as indicated above. One patient died, representing a mortality rate of 2.5% for the whole cohort.

## Discussion

Adhesions are a major burden for patients' well-being and a contributor to the surgeons' frustration. In a cohort of 12,584 patients undergoing open abdominal surgery, 33% of patients were readmitted a mean of 2.2 times in the subsequent 10 years for possibly adhesion-related complications. 7 A severe complication is intestinal obstruction which has a mortality rate of up to 15%. 10 In addition to their clinical evidence, adhesions are a major cost factor in health care. 17 If adhesions necessitate surgery, they reoccur in 55 to 100% of patients postsurgically, with an average of approximately 85%. 5 The predominant locations of reoccurrence are the former sites of adhesiolysis. 12 Upon reformation, adhesions can be more severe requiring additional surgeries. 19 36 37 38 The introduction of laparoscopy has been reported to have reduced the incidence and severity of adhesion formation as compared with laparotomy. 39 40 41 However, neither complications nor costs have been reduced substantially by introducing laparoscopic surgery. 42 Since adhesions have a substantial clinical impact, much effort has been put into developing effective adhesion prevention devices in the last decades. 43 They act as barriers separating impaired neighboring peritoneum until healing of the mesothelial surfaces is completed. However, Ahmad et al 44 did not find conclusive evidence for effectiveness of the long-standing marketed adhesion barriers. As bleeding can contribute to adhesion formation and since many devices for adhesion prevention cannot be used in the presence of blood, we decided to use 4DF, a medical device with properties for both adhesion prevention and hemostasis. Adhesive SBO as in our series still is a challenge with uncertain early and late outcomes. To the best of our knowledge, no study has been published describing the use of 4DF gel for such severe clinical condition or in another general surgery cohort except for a case series just published by Blumhardt et al. 31

The high median adhesion severity and extent scores during the first operation combined with the long operation times and high rates of conversion to and open surgery from the beginning underline that the patients of the present study form a cohort with severest diseases. Nevertheless, only one patient died during hospitalization. One patient with persistent, adhesive obstruction and massive firm peritoneal adhesions even felt so well after surgery that he requested an early discharge. Furthermore, with 4DF significant reductions of adhesion severity and adhesion extent scores were detected in redo-surgeries. The comparison of postoperative laboratory parameters of the patients treated with 4DF and patients without treatment indicated no difference between both groups in most parameters. The significantly lower mean postoperative hemoglobin and hematocrit levels in the 4DF group indicate that the severity of the surgical interventions was higher and accordingly more blood loss occurred. The higher severity of the interventions is underlined by a significantly longer operation time in the 4DF group. A postoperative rise of the CRP level after application of 4DF was also indicated in the instructions for use. 45 In accordance with the instructions for use, the CRP level rise was temporary and the mean postoperative leukocyte levels and body temperatures were not elevated in the 4DF group as compared with the untreated patients.

Severe adhesions and a long duration of adhesiolysis are known to be predictors of a higher complication incidence. 14 Our results are in line with this observation since none of the patients with an operation time of ≤ 1-hour experienced postoperative complications. Our conversion rate of 25% also corresponds to published results of SBO management which reported conversion rates of 26 to 54%. 9 All complications noted are common after this type of surgery as described by Nakamura et al 46 and, therefore, could not be ascribed to the use of 4DF gel. Furthermore, the infections observed in two patients were caused by the skin bacteria *S. aureus* , likely originating from the environment.

During the follow-up, none of the patients experienced a recurrent obstruction episode. This is a remarkable result considering that recurrence rates range between 10 and 53% in other studies with follow-ups between 7 months and 15 years. 13 46 47 Furthermore, open surgery and conversion to open surgery are risk factors for recurrent obstructions. 46 Nevertheless, despite our high incidence of primary open surgery and conversion, there were no obstructive episodes during the follow-up periods.

The proof of efficacy of adhesion prevention devices remains difficult. The absence of clinical symptoms does not rule out the presence of adhesions. In our cohort, eight patients had to undergo reinterventions for different pathologies between postoperative days 1 and 155. The findings in these patients are meaningful since it is known that reformation of peritoneal adhesions develops within the first few days after surgery and that peritoneal recovery occurs within 3 to 5 days independent of the size of the peritoneal damage. 48 Accordingly, if peritoneal defects have healed in the first postoperative days without developing adhesions, it can be expected that they remain free of adhesions in the later course. In none of our patients with early redo-surgery adhesions were detected. Therefore, early adhesion prevention can be rated as highly successful. This is remarkable since the conversion rate, percentage of open surgery, and duration of the operation, all indicators for the severity of the adhesion disease, 14 46 were high and recurrence rates are up to 100% according to Vrijland et al. 11 The finding of adhesion formation in the patient with wound dehiscence during hospitalization deserves special consideration. He was free of adhesions on postoperative days 13 and 21 due to efficient adhesion prevention measures. For the adhesion formation found in this patient on postoperative day 25, the lack of 4DF application in the previous reintervention, as well as inflammatory fasciitis were likely causative. Inflammation is known to induce an imbalance between fibrinolysis and fibrin deposition in favor of deposition, 49 which is a key factor in the development of adhesive bands and agglutination. 38

In summary, all but one patients were free of adhesions in redo surgeries and none of the patients had recurrent obstructive episodes following treatment with 4DF gel. Furthermore, no remnants of the product were found during the reinterventions and no 4DF related complications occurred, indicating a very good biocompatibility and tolerability of the product like reported earlier. 23 26 50 51

## Conclusion

In this cohort with adhesive SBO 4DryField PH gel application was save. In combination with adhesiolysis, it seems to be effective in preventing peritoneal adhesions and recurrence of obstructive episodes. Although one cannot distinguish the influence of 4DryField PH from the effect of adhesiolysis alone, considering the severity of the disease and predisposition for adhesion formation in these patients, adhesion prophylaxis with the polysaccharide gel can be rated as highly successful, evidenced by a significant reduction of adhesion severity and adhesion extent scores in reinterventions. Its application is a promising treatment for prevention of adhesive SBO and should be investigated in larger prospective randomized trials.
